# Differential effects of levodopa on social cognition in people with Parkinson's disease

**DOI:** 10.1177/1877718X261435304

**Published:** 2026-04-29

**Authors:** Teodor Danaila, Élise Métereau, Hélène Klinger, Adélaide Jaulent, Océane Porte, Léon Tremblay, Paul Jaulent, Chloé Laurencin, Stéphane Prange, Stéphane Thobois

**Affiliations:** 1 Service de Neurologie C, Centre Expert Parkinson, NS-Park network, Hôpital Neurologique Pierre Wertheimer, Hospices Civils de Lyon, France; 2 PATHPARK team, Lyon Neuroscience Research Center (CNRL), INSERM U1028, CNRS UMR5292, Lyon, France; 3 Intensive Neurological Care Unit, Hôpital Neurologique Pierre Wertheimer, Hospices Civils de Lyon, Lyon, France; 4 Centre de Neurosciences Cognitives - UMR 5229, CNRS, Université Lyon 1, Lyon, France; 5 Faculté de Médecine et de Maïeutique Lyon Sud Charles Merieux, Université Claude Bernard Lyon 1, Lyon, France

**Keywords:** social cognition, theory of mind, emotional resonance, Parkinson's disease, levodopa

## Abstract

Social cognition impairment is a frequent non-motor feature of Parkinson's disease. While dopaminergic therapy modulates motor symptoms, its effects on social cognition remain incompletely understood. We investigated the effects of acute levodopa administration on cognitive and affective Theory of Mind, as well as on emotional resonance to dynamic whole-body social interactions, in 36 people with Parkinson's disease with motor fluctuations and 14 matched healthy controls. Social cognition was assessed using the Mini-Social Cognition and Emotional Assessment (Mini-SEA) and a point-light display task indexing emotional resonance through emotional valence ratings. Patients were evaluated in OFF and ON states during an acute dopaminergic challenge performed according to the CAPSIT-PD protocol, with responsiveness defined as an improvement greater than 50% on the MDS-UPDRS part III. Compared with healthy controls, patients showed impaired cognitive Theory of Mind performance, particularly on the faux pas subtest (p = 0.0001), while affective Theory of Mind based on facial emotion recognition was preserved. Acute levodopa did not improve cognitive or affective Theory of Mind (faux pas OFF vs ON, p = 0.7049). In contrast, emotional resonance was impaired in the OFF state and selectively improved in the ON state, with increased ratings of positive (p = 0.0035) and negative (p = 0.0387) emotional valence. These findings demonstrate a dissociation between Theory of Mind and emotional resonance in Parkinson's disease and show that acute levodopa selectively modulates emotional resonance without restoring Theory of Mind abilities.

## Plain language summary

How levodopa changes emotional responses to social situations in Parkinson's disease

People with Parkinson's disease often experience difficulties in social situations, including problems understanding other people's emotions. These difficulties are not only related to movement symptoms but can also affect everyday interactions and quality of life. In this study, we examined how levodopa, a commonly used medication for Parkinson's disease, influences different aspects of social understanding. We studied people with Parkinson's disease who experience motor fluctuations, meaning that their symptoms vary depending on whether their medication is working or wearing off. Their performance was compared with that of healthy individuals. Participants completed tasks measuring their ability to understand other people's intentions and emotions. They also evaluated short videos showing interactions between two individuals represented by moving points of light, a technique that captures body movements without showing faces or detailed features. Participants rated the emotional tone of these interactions. People with Parkinson's disease showed difficulties understanding complex social situations, and these difficulties did not improve after taking levodopa. However, when judging emotional interactions expressed through body movements, patients showed clearer emotional responses after taking levodopa compared with when they were off medication.

## Introduction

Social cognition can be defined as the set of mental processes and cognitive abilities that enable individuals to perceive, interpret, and respond to the behaviors, intentions, emotions, and social signals of others. Social cognition supports the understanding of social interactions, from overt actions performed by others to more subtle cues such as facial expressions, tone of voice, and body language. In addition, it underpins the capacity to navigate and maintain interpersonal social relationships. The key components of social cognition are: 1. Social perception, which corresponds to the processing of social cues, including facial expressions, vocal tones, and body language, to interpret social information accurately, 2. Emotion recognition and the processing or the ability to recognize, interpret, and respond appropriately to the emotional expressions of others, which is essential for empathy and social responsiveness, 3. Empathy or the capacity to feel and understand the emotions of others, often divided into cognitive empathy (understanding another's emotional state) and affective empathy (emotionally resonating with them), 4. Theory of Mind (ToM), which refers to the mentalization capacity necessary to attribute mental states—such as beliefs, desires, and intentions—to oneself and others, recognizing that others have perspectives different from one's own, 5. Social decision-making and judgment, which include the evaluation and interpretation of social interactions, norms, and values that inform decision-making in social contexts and finally 6. Self-referential thinking or the process of relating social information to oneself, which is important for understanding one's role in social interactions and forming a social identity.^1^

ToM^2,3^ is a multifaceted cognitive ability that can be further divided into two main components: Cognitive ToM and Affective ToM. The first component is the ability to infer beliefs, intentions, and knowledge states of others, and is often regarded as a “cold” cognitive function as it requires perspective-taking and mentalizing without necessarily involving emotional processing.^4^ The second component involves understanding and empathizing with others’ emotions, often seen as a “hotter” aspect of ToM due to its reliance on emotional resonance and empathy.

The mirror neurons model (MNM) is presumed to play a critical role for social cognition.^5^ Mirror neurons are activated both when individuals perform a specific action and when they observe someone else performing similar action. This unique feature makes mirror neurons essential for understanding the actions, intentions, and emotions of others by simulating or “mirroring” those experiences internally. This system simulates an observed action, by translating it into an internal motor representation. This system is not only involved in body motion, but also in emotional processes, making it a key player in social interaction.^6^ There is a conceptual overlap between mirror neurons and ToM, particularly via their shared goal of understanding others. However, their mechanisms and roles are distinct, with mirror neurons contributing to automatic simulation and ToM enabling higher-order reasoning. Understanding their interaction is key to comprehending the full spectrum of social cognition.

In people with Parkinson's disease (PPD), several studies revealed that social cognition tasks, such as mind-reading, facial expression recognition (« affective ToM») and decision-making (« cognitive ToM») are impaired.^7–10^

While the expression of emotions is often studied through facial expression, as examined in the classic Ekman 60 Faces Test, human body movements also play a crucial role in conveying social learning, past experiences and current emotions. Few studies have assessed, in PPD, the ability to understand others emotions expressed through their social whole-body interactions.^11,12^ Therefore, identifying deficits of this capacity could provide valuable insights to understand social interactions difficulties experienced by PPD. Kloeters et al.^11^ found that PPD showed impaired perception of human movements in OFF state compared to healthy individuals, particularly in recognizing goal-directed or transitive movements (involving objects). The authors suggest that this deficit may be directly related to the fine motor execution impairments, secondary to PD, supporting the idea that PPD, who are impaired to perform accurate movements may also struggle to perceive and interpret others’ motor actions, as predicted by the MNM. More recently, Bellot et al.^12^ investigated how emotional valences were perceived by PPD, who visualized short movies depicting emotional interactions between two human characters using the Point Light Display technique (PLD).^13,14^ When compared to healthy controls, PPD showed significantly reduced emotional ratings of the scenes, despite a good understanding of the motor interactions.

Beyond Parkinson's disease PLD paradigms have been widely used in psychiatry to characterize impairments in processing biological motion and social interactions. In autism spectrum disorders, several studies have shown altered recognition of emotional body movements and reduced sensitivity to biological motion cues,^6^ suggesting atypical functioning of the mirror neuron system and mentalizing networks. In schizophrenia, PLD tasks consistently reveal deficits in interpreting goal-directed actions and emotional interactions, as well as impaired integration of kinematic cues into coherent social meaning. Tomlinson et al.^15^ demonstrated that individuals with schizophrenia exhibit marked difficulties in decoding emotional valence from dynamic PLD scenes, supporting the idea of disrupted embodied simulation mechanisms. PLD abnormalities have also been reported in mood disorders, including major depression,^16^ where blunted emotional reactivity to PLD scenes mirrors the reduced affective engagement commonly observed clinically.

Dopaminergic degeneration is responsible for most motor signs and many emotional dysfunctions in PPD (apathy, anxiety, depression).^17^ However, the impact of dopaminergic treatments on social cognition mediated by body movements remains unclear, in PPD who experience fluctuations. Most studies have examined ToM and related aspects in a non-controlled ON state, despite the variability of chronic dopaminergic treatment throughout the day.^18^

In this study, we assessed both affective and cognitive ToM, as well as emotion recognition conveyed by motor interactions between two human silhouettes during a specific PLD task and investigated the role of dopaminergic treatment in OFF and ON states. We hypothesized that the perception of emotions conveyed by social interactions would be impaired in PPD and might be restored by dopaminergic therapy.

## Methods

In this study (NCT03004573), patients with Parkinson's disease and age- and sex-matched healthy controls were enrolled. Inclusion criteria for PPD were: age between 40 and 70 years; presence of motor fluctuations; good levodopa responsiveness (improvement of MDS-UPDRS III score > 50% after levodopa challenge); and no significant cognitive decline, as measured by standard psychometric tests usually used for the selection of PPD candidates for deep brain stimulation (DBS). The study received approval from the ANSM (Agence nationale de sécurité du médicament et des produits de santé) and the appropriate ethics committee (Comité de Protection des Personnes France Sud-Est III in 2016, revised by Comité de Protection des Personnes France Nord Ouest II in 2022). Written informed consent was obtained from all participants prior to testing, in accordance with the Declaration of Helsinki.

A comprehensive motor assessment (MDS-UPDRS part III) was conducted in both OFF and ON states using an acute dopaminergic challenge performed according to the Core Assessment Program for Surgical Interventional Therapies in Parkinson's Disease (CAPSIT-PD).^19^ All antiparkinsonian medications were withdrawn for at least 12 h to obtain a defined-OFF condition. Patients then received their regular morning dose of oral levodopa. Motor assessment in the defined-ON condition was performed at the time point at which both the patient and the investigator agreed that the clinical benefit was maximal. In accordance with current clinical practice, dopaminergic responsiveness was defined as an improvement greater than 50% on the MDS-UPDRS part III. The use of a standardized CAPSIT-PD-based dopaminergic challenge ensured a robust and reproducible assessment of acute levodopa effects across participants.

Neuropsychiatric and cognitive assessments were conducted in the ON state, including evaluations of anxiety (Hamilton anxiety scale, HAS), depression (Hamilton depression scale, HDS), apathy (Lille Apathy Rating Scale), cognitive functions (MoCA -Montreal Cognitive Assessment, Grober and Buschke 16-items free and cued recall test, semantic verbal fluency test, Boston Naming Test, Clock drawing and copy, visuospatial judgment Benton test), as well as quality of life using the 39-item Parkinson's disease questionnaire (PDQ-39). For Theory of Mind (ToM) assessment, we used Mini-SEA^20,21^ a validated scale comprising two subtests: facial emotion recognition^22^ and a modified faux pas test.^23^ Then, all participants were asked to judge emotional scenes depicting whole-body movements using specific PLD tasks described previously.^12^ All participants visualized silent movies of 3 s each and were asked immediately after to rate the emotional valence on a subjective scale from −5 to +5 for each scene depicting motor interactions between two facing human characters using the PLD technique (PsyScope X application - https://cbclab-online.upf.edu/rico/psyscope/).^24^ A total of fifteen scenes^25^ were presented twice each, in random order, on a 15-inch MacBook screen. Of the 15 scenes, 5 depicted negative emotions (e.g., sadness, dispute, anger), 5 depicted positive emotions (e.g., joy, dancing, laughter), and 5 were neutral (e.g., sitting, saying hello, raising arms) Patients received no feedback following their ratings. For each patient, we calculated the average scores for the films with negative, neutral, and positive valences, with results ranging from −5 to +5. For both the mini-SEA and the PLD whole-body emotional task, PPD were evaluated in the OFF and ON states during the levodopa acute challenge test. To prevent a learning effect, we randomized the order of ON and OFF evaluation, so that half of the PD patients were evaluated in the OFF state and the other half in the ON state first.

**Figure 1.**
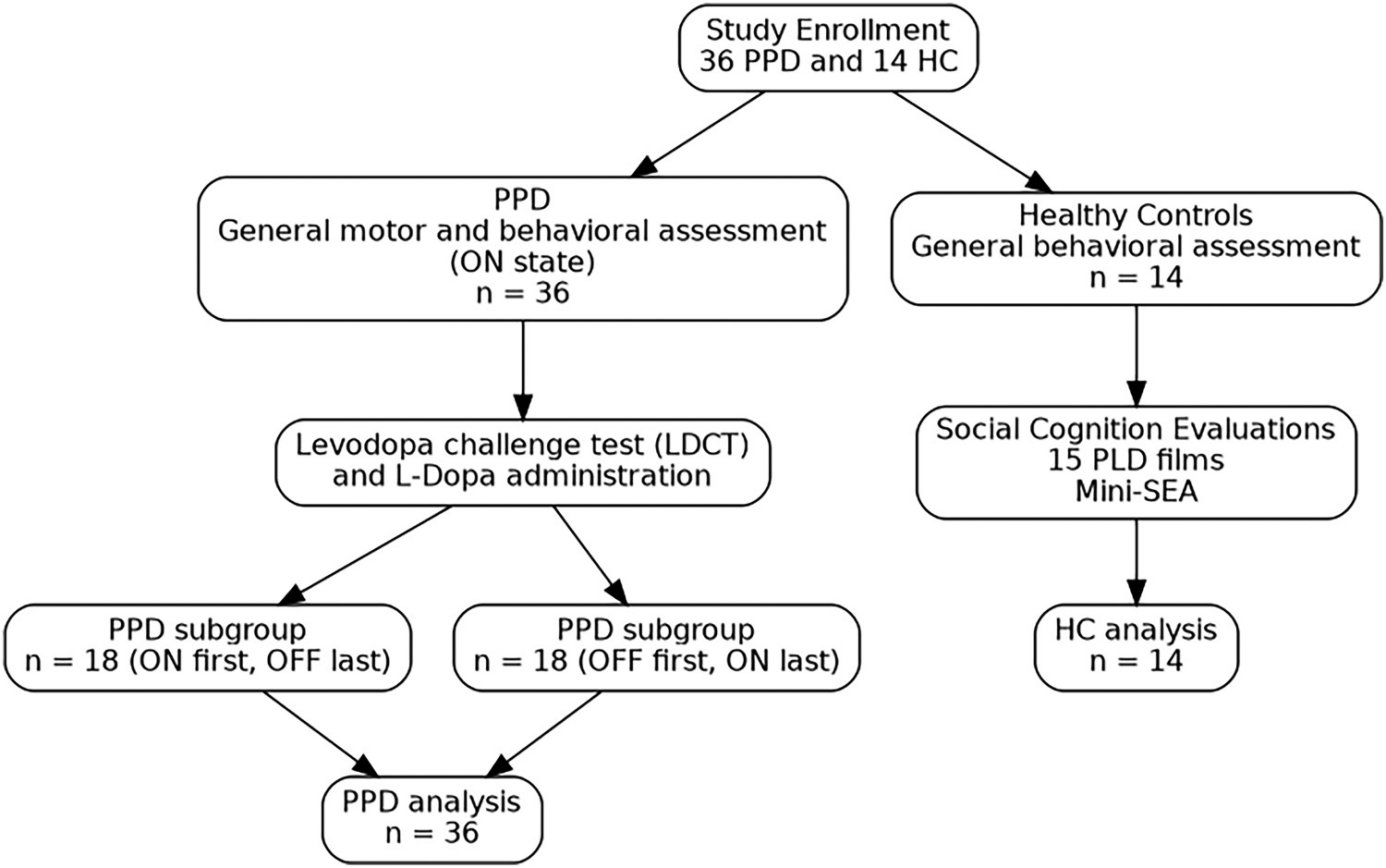
The study flowchart. PPD : People with Parkinson's Disease, HC : Healthy Controls.

The study flowchart is presented in Figure 1.

## Data analysis

Sex and gender considerations. Sex was recorded for all participants. The study was not powered for sex-stratified analyses; therefore, sex was handled descriptively, and any sex-related analyses were pre-specified as non-inferential, in accordance with SAGER recommendations. Exploratory checks for potential sex trends were conducted for the main outcomes and are reported below.

Descriptive statistics were used for group descriptions, and quantitative variables were summarized using medians ± interquartile range (IQR). Comparisons with HC were made using the Mann-Whitney U test for two independent groups. Within the PDD group comparisons, Wilcoxon's test for paired samples was applied to assess the effects of dopaminergic challenge (OFF versus ON state). Correlations between the PLD tasks and ToM, motor, and behavioral scores were evaluated using Spearman's rank correlation coefficient. The significance threshold for all tests was set to p < 0.05. Statistical analyses were performed using R software version 4.4.1 (R Core Team, www.R-project.org).

## Results

36 patients with fluctuating PD and without cognitive impairment and 14 healthy controls were included. All patients were on chronic dopaminergic treatment, including levodopa monotherapy or a combination of levodopa and dopamine agonists. No patient was treated exclusively with dopamine agonists. 10 patients were receiving a COMT inhibitor, and 12 were on amantadine. The mean L-Dopa equivalent daily dose (LEDD) was 1367.50 mg (± 796.48 mg). All patients had global cognition scores within normative ranges and demonstrated a motor response greater than 50% during levodopa challenge. Among them, five individuals showed mild apathy (LARS > −21), five had mild depression (HAM-D score between 8 and 14), and three had mild anxiety (HAM-A score between 8 and 14). Clinical characteristics are presented in Table 1. In the following sections, OFF and ON states refer to the defined-OFF and defined-ON conditions obtained during the CAPSIT-PD levodopa challenge.

**Table 1.** Demographic and clinical characteristics of the participants. Median ± IQR are reported. Statistical analyses were performed using Wilcoxon Mann–Whitney tests and Fisher tests for sex.

	PPD	HC	Comparison p-value
Number	36	14	
Age	59.00 ± 11.25 [44 ; 69]	62.50 ± 6.25 [52 ; 69]	0.2888
Sex ratio (F/M)	10/26	5/9	
Disease duration (y)	10.00 ± 6.25 [4 ; 18]		
MDS-UPDRS III OFF medication	34.00 ± 14.50 [20 ; 85]		
MDS-UPDR III ON medication	9.50 ± 9.00 [2 ; 48]		
LEDD total (mg)	1367.50 ± 796.48 [650 ; 2800]		
LEDD DA agonists (mg)	150.00 ± 324.38 [0 ; 1430]		
MoCA	29.00 ± 2.00 [24 ; 30]	29.00 ± 2.00 [27 ; 30]	0.7878
Depression score (HAM-D)	4.00 ± 4.00 [0 ; 11]	3.00 ± 3.75 [0 ; 7]	0.1051
Anxiety score (HAM-A)	2.50 ± 4.00 [0 ; 12]	2.00 ± 2.75 [0 ; 8]	0.7023
Apathy score (LARS)	−27.26 ± 4.93 [−29 ; 7]	−29.50 ± 4.75 [−35 ; -25]	0.1452

PPD: People with Parkinson's disease, HC: Healthy Controls.

Exploratory analyses comparing patients treated with levodopa alone versus those receiving combined levodopa and dopamine agonist therapy did not reveal any significant differences in Mini-SEA subscores or PLD emotional ratings, in either OFF or ON states. Exploratory (non-inferential) checks did not suggest sex-related trends on Mini-SEA subscores or PLD emotional ratings in either OFF or ON states.

### Impact of PD and levodopa on Theory of Mind evaluation

**Figure 2.**
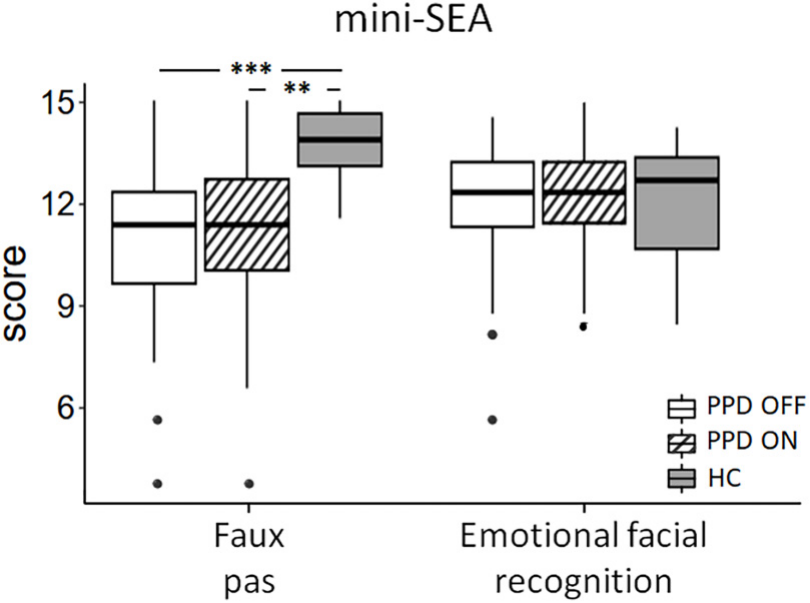
Between-group comparison of the cognitive and affective ToM using the mini-SEA evaluation in HC and PPD in the OFF and ON states.

Regarding ToM, the Mini-SEA showed that PPD in OFF condition performed significantly worse on the faux pas subtest (median ± IQR =11.44 ± 3.00) compared to HC (13.88 ± 1.41; p = 0.0001), while no significant difference was observed between the ON (11.07 ± 3.10) and OFF states in PDD (p = 0.7049; Figure 2). By contrast, performance for the emotional facial recognition test was similar in PPD in OFF condition (12.43 ± 1.29) and HC (12.65 ± 2.37, p = 0.8362; Figure 2), without difference in the OFF and ON (12.43 ± 1.72) conditions in PPD (p = 0.6123). Accordingly, the total Mini-SEA score for individuals with PD OFF levodopa (23.63 ± 3.59) was lower than that of healthy controls (25.42 ± 2.78, p = 0.0029), with no significant change in performance between the OFF and ON (23.60 ± 3.64) states after administering an oral levodopa dose (p = 0.4561).

We found no statistical correlation between the low mini-SEA scores and patients’ age, disease duration, or motor impairment. Years of education were calculated as the total number of completed years of formal schooling. Exploratory analyses comparing patients with lower versus higher educational levels did not reveal any significant differences in faux pas performance, emotional facial recognition, or total Mini-SEA scores, in either OFF or ON states. However, we observed a strong association between poorer faux pas subtest performance and lower scores in verbal fluency (p = 0.0065) and free recall of 16 words (p = 0.0492).

### Influence of PD and levodopa on emotional valences of the PLD social motor interactions

**Figure 3.**
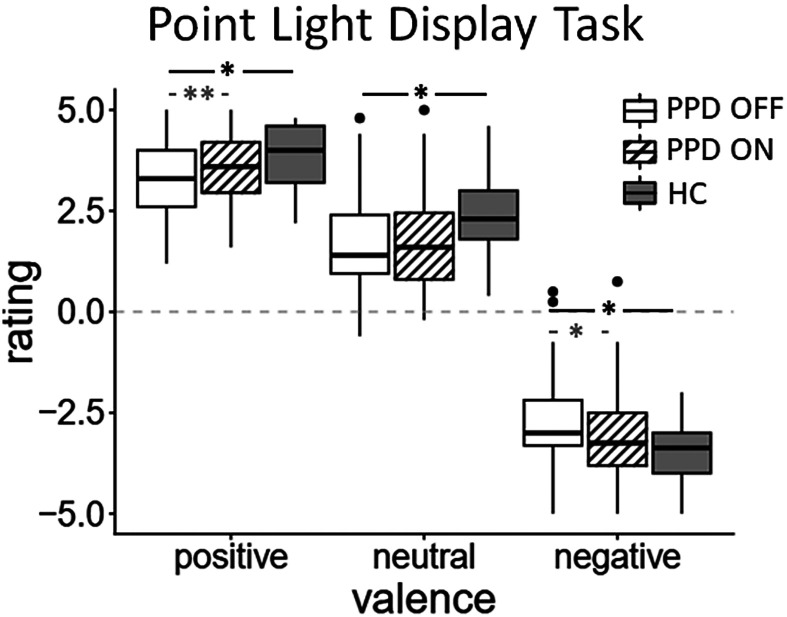
Between-group comparison of the emotional ratings for positive, neutral and negative scenes using the PLD whole-body emotional task in HC and PPD in the OFF and ON states.

Positive, negative, and neutral PLD whole-body emotional scenes were presented to HC, and PPD in the OFF and ON states. For positive emotional content scenes, PPD rated the absolute value of emotional valence significantly lower in the OFF state (median ± IQR = 3.00 ± 1.45) in comparison to the ON state (3.60 ± 1.25, p = 0.0035) and compared to healthy controls (4.00 ± 1.30, p = 0.0296). Similarly, for negative emotional content scenes PPD also rated their absolute value significantly lower in the OFF state (−2.88 ± 0.81) than in the ON state (−3.13 ± 1.31, p = 0.0387) and compared to healthy controls (−3.38 ± 1.00, p = 0.0252). By contrast, neutral emotional content scenes were rated similarly by PPD in the OFF (1.40 ± 1.60) and ON state (1.60 ± 1.80, p = 0.1056) and slightly higher to those of healthy controls (2.30 ± 1.15, p = 0.0339), as illustrated in Figure 3.

No significant differences were observed between the ratings PLD emotional scenes of PD participants in the ON state and those of healthy controls, for either positive (p = 0.3918) or negative scenes (p = 0.2496).

Change of rating between the OFF and ON states did not correlate with patient age, disease duration or cognitive scores, mini-SEA scores, or PDQ-39 scores. Notably, there was no correlation between the severity of motor impairment, as measured by the MDS-UPDRS III score, and the variations of the emotional ratings of the PLD scenes. Although a small subset of patients presented mild apathy, no association was found between apathy severity and Theory of Mind performance, PLD emotional ratings, or levodopa-induced changes, suggesting that apathy did not act as a confounding factor in the present study.

## Discussion

To the best of our knowledge, this study is the first to examine the effects of an acute levodopa challenge on social cognition in PPD, specifically addressing both affective and cognitive ToM, as well as emotional valences conveyed through whole-body emotional interactions via a PLD task. We demonstrate that individuals with PPD without cognitive impairment show deficits in judging whole-body emotional scenes, a capacity that is modulated by levodopa. Levodopa restored emotional responses to levels closer to those observed in healthy controls for both positive and negative interactions (e.g., happiness, joy, anger, fear, sadness). By contrast, levodopa did not restore affective or cognitive ToM.

Our findings confirm that the ability to evaluate the emotional valence of whole-body social interactions is impaired in PPD in the OFF state, consistent with previous studies using similar PLD tasks.^11,12^ In contrast, the recognition of emotions conveyed via facial expressions (affective ToM) remained preserved in PPD.

Recent neuroimaging evidence further supports this distinction by suggesting the existence of partially dissociable mirror neuron networks for social versus non-social actions, which may be differentially affected in Parkinson's disease and contribute to altered processing of dynamic whole-body social interactions, as assessed by PLD paradigms.^26^ This aligns with previous behavioral studies^18^ suggesting the existence of two distinct but complementary emotional recognition systems: one driven by motor interactions (as posited by the MNM), and the other based on facial expression recognition. This distinction may reflect differences in the pathways underpinning social behavior perception, which extends beyond facial and mouth expressions. Specifically, separate anatomical pathways are thought to mediate mirror neuron networks involved in social interactions conveyed through facial versus limb motor actions.^27^ Indeed, while facial expression processing appears to engage the ventrolateral prefrontal cortex and limbic structures, limb motor actions rely heavily on visual and sensorimotor networks. Reduced activation in mirror neuron areas, particularly in the opercular region of the inferior frontal gyrus,^28^ has been associated with poorer performance in recognizing facial emotions in PPD.

We hypothesize that Parkinson's disease differentially affects mirror neuron networks, contributing to blunted emotional ratings for dynamic whole-body emotional scenes in the OFF state compared to the ON state and healthy controls.

Additionally, our results indicate that acute levodopa administration enhances emotional responses. This suggests that levodopa causally restores the ability to perceive whole-body emotional content, independent of motor impairment severity, as no association was found between motor symptoms and emotional blunting. Importantly, the Ekman facial recognition task, which does not involve motor activity, showed no evidence of dopaminergic modulation. Thus, our findings demonstrate that impairments in perceiving emotions carried out by human interactions primarily depend on dopaminergic modulation and are related to the intensity of emotions conveyed by body movements.

In contrast to Bellot et al.,^12^ our findings suggest that dopaminergic treatment improves and restores the ability to appropriately judge emotional motor scenes in a controlled ON-state. Furthermore, we observed that levodopa specifically modulates social cognition involving emotional judgment without significantly affecting affective or cognitive ToM. Although dopamine agonists more selectively target mesolimbic pathways, exploratory analyses comparing patients treated with levodopa alone versus combined levodopa–dopamine agonist therapy did not reveal differential effects on Theory of Mind performance or PLD emotional ratings, suggesting that the observed effects are primarily driven by acute levodopa modulation. We propose that levodopa may enhance mesolimbic pathway functioning and limbic cortico-subcortical loops, improving the processing of both positive and negative experiences and the interpretation of motor interactions. This hypothesis aligns with findings on the ventral tegmental area (VTA),^29^ where dopaminergic neurons are known to process both aversive and reward-related stimuli, as demonstrated in freely behaving mice.^30^ This hypothesis is supported by electrophysiological studies involving local field potential recordings of the subthalamic nucleus in PPD patients with bilateral subthalamic DBS. These studies have shown that dopamine facilitates the processing of pleasant stimuli while suppressing activation during unpleasant stimuli. Notably, this effect, reflected by desynchronization in the alpha frequency band, is reversed in the OFF-medication state.^31^

The association between poorer faux pas performance, reduced verbal fluency, and lower free recall suggests shared fronto-subcortical mechanisms underlying cognitive Theory of Mind impairment in Parkinson's disease, consistent with executive and strategic retrieval dysfunction rather than a purely social cognitive deficit. These deficits were not improved by levodopa, consistent with previous findings in advanced or late-onset PD.^18^ Recent studies^32–34^ have also shown cognitive ToM impairments in early-stage PD, linking them to subtle cognitive dysfunctions, such as reduced verbal fluency and recall. Longitudinal follow-up may help determine whether early cognitive ToM impairments predict a higher risk of Parkinson's disease dementia. Therefore, cognitive ToM impairment, notably with the faux pas subtest of the Mini-SEA could be part of routine cognitive assessment for a better sensitivity than existing psychometric assessments to detect early decline.

In contrast, we found that affective ToM (facial expression recognition) was preserved in fluctuating PPD. However, this remains debated due to variability in study populations and tasks.^32–34^ For example, Dodich et al.^35^ reported impaired facial emotional recognition in PD with mild cognitive impairment (MCI) and even in cognitively unimpaired PPD, though the latter group had significantly lower MoCA scores compared to our study cohort and were generally below the accepted normative threshold for this cognitive test. Similarly, Czernecki et al.^34^ observed early affective ToM impairments in PD patients with a disease duration of less than 24 months, potentially reflecting population heterogeneity and differences in educational background or cognitive reserve.^36^ Additionally chronic dopaminergic treatment may partially stabilize affective ToM abilities by facilitating adaptations in mesolimbic and prefrontal circuits.^10^

DBS of the subthalamic nucleus has been also shown to modulate emotional processing and certain aspects of social cognition through its effects on limbic fronto-striatal circuits.^37^ Building on the present findings, we plan to investigate these DBS-related effects using the same controlled ON/OFF DBS methodology in a future study.

This study has some limitations, including moderate sample size and heterogeneity in cognitive reserve and chronic dopaminergic regimens among participants. However, as a pilot study, it was designed to explore the effects of an acute, controlled oral levodopa challenge on emotional perception in fluctuating PPD without cognitive decline. Furthermore, randomizing the order of OFF and ON conditions and minimizing feedback reduced potential test-retest effects. However, PLD stimuli remain highly schematic and artificial, lacking fine-grained contextual, facial, and kinematic cues present in real social interactions, which may limit the ecological validity of the emotional judgments obtained.

Although we reported sex distribution, the present study was underpowered to test sex-related effects on social cognition or on levodopa modulation. Considering prior evidence that sex may influence Parkinson's disease features and social cognition, larger cohorts should prospectively address sex and gender as biological and sociocultural variables of interest.

In conclusion, levodopa selectively modulates social cognition by restoring the ability to judge emotions conveyed by whole-body movements performed by others, enhancing both positive and negative judgments of motor social interactions in PPD without cognitive impairment, independently of their motor impairment. Future studies combining behavioral and brain imaging approaches are warranted to further investigate emotional blunting, its impact on daily functioning in PD, and the roles of other dopaminergic treatments and DBS.
